# Microstructural and functional analysis of PLA-based biofilm reinforced with *Sechium edule*

**DOI:** 10.1016/j.fochx.2025.103130

**Published:** 2025-10-03

**Authors:** Kaliraj Medadurai, Senthil Maharaj Kennedy, Jegan Balasubramani, Selvi Shanmugavelayutham

**Affiliations:** a Department of Mechanical Engineering, AAA College of Engineering and Technology, Sivakasi 626005, Tamilnadu, India; b Department of Mechanical Engineering, Velammal College of Engineering and Technology, Madurai 625009, Tamilnadu, India; c Department of Information Technology, Dr. Sivanthi Aditanar College of Engineering, Tiruchendur 628215, Tamilnadu, India

**Keywords:** PLA biofilm, *Sechium edule*, Biodegradable composites, Green packaging, Natural reinforcement

## Abstract

This paper details the development and characterization of biodegradable composite biofilms composed of polylactic acid (PLA) reinforced with *Sechium edule* peel powder, a sustainable agricultural by-product. The biofilms were produced using solvent casting with different filler loadings (0–20 wt%) and assessed for mechanical, morphological, thermal, and environmental characteristics. The results demonstrated a notable increase in tensile strength (up to 38.5 MPa at 10 % SE) and modulus, as well as enhanced biodegradability and water absorption capacity at elevated reinforcement levels. FTIR validated the successful integration of lignocellulosic functional groups, whilst FESEM and AFM studies demonstrated uniform filler distribution up to 15 %. Biodegradability assessments demonstrated a weight reduction of up to 9.7 % within 7 days under regulated settings, signifying expedited degradation. The incorporation of *Sechium edule* enhances the functional efficacy of PLA while also advancing circular bioeconomy objectives. These results underscore the composite's promise for sustainable packaging, agricultural mulch films, and low-impact biomedical applications, with significant prospects for industrial scalability.

## Highlights

• *Sechium edule* peel powder effectively reinforced PLA biofilms, enhancing biodegradability and hydrophilicity.
• FTIR and FESEM confirmed successful filler incorporation and improved interfacial morphology.
• Biofilms show potential for sustainable packaging and agricultural applications.

## 1 Introduction

The extensive use of petroleum-based plastics in packaging and related industries has created a severe environmental burden, as these materials often accumulate in landfills and marine environments, where breakdown occurs at an exceedingly slow rate or not at all (Pawle et al., 2025; Zhao et al., 2025). This alarming scenario has accelerated the search for sustainable alternatives such as green packaging, which emphasizes the use of biodegradable, renewable, and eco-friendly materials designed to minimize environmental impact throughout their life cycle (Drechsel, Kracklauer, Menrad, & Decker, 2025; Nguyen Quoc, Nghiem Phuc, & Duong, 2025). Among the candidates, polylactic acid (PLA) has gained prominence as a biodegradable polymer derived from renewable resources that can undergo microbial decomposition in composting environments (Asmat-Campos, Rojas, Carreño-Ortega, & Raquel-Checca, 2025; Tutek, Rosiak, Kałużna-Czaplińska, & Masek, 2024). However, the intrinsic brittleness, slow degradation rate, and hydrophobic nature of pure PLA limit its widespread application in areas requiring rapid disintegration or effective moisture interaction. To overcome these limitations and improve environmental performance, researchers have explored the incorporation of natural fillers into PLA matrices. These bio-fillers not only enhance disintegration and water affinity but also contribute to lowering the overall carbon footprint of the composite materials. Thus, the development of PLA-based composites reinforced with natural fillers represents a timely and necessary step toward advancing green packaging solutions to address the pressing environmental challenges of the 21st century (Chiloeches et al., 2022; Mai et al., 2024).

Despite its biodegradability and derivation from renewable resources, pure polylactic acid (PLA) presents certain constraints that impede its efficacy in practical packaging and biological applications. A significant disadvantage is its intrinsic brittleness and low elongation at break, generally approximately 6 %, which limits its flexibility and toughness in dynamic applications. Moreover, PLA exhibits a very low heat resistance, with a glass transition temperature of between 55 and 60 °C, rendering it inappropriate for applications requiring increased temperatures, such as hot-fill packing (DeStefano, Khan, & Tabada, 2020; Hussain, Khan, Shafiq, & Abbas, 2024). Its inadequate moisture resistance and limited water vapour barrier qualities further diminish its efficacy in preserving perishable items. In biomedical contexts, although PLA is biocompatible and sanctioned for specific medical devices, its protracted breakdown rate—spanning 6 to 24 months under physiological conditions—can pose challenges in applications necessitating expedited resorption, such as temporary scaffolds or drug delivery systems. Furthermore, the hydrophobic characteristics of clean PLA impede cell attachment and proliferation, which are essential in tissue engineering. These constraints require structural and compositional alterations, including the integration of hydrophilic and biodegradable natural fillers, to customize the properties of PLA for wider, high-performance applications in sustainable packaging and biomedical sectors (Jacob et al., 2024; Ramezani Dana & Ebrahimi, 2023).

Natural reinforcements have emerged as promising sustainable additions for improving the properties of biodegradable polymers, providing an environmentally benign alternative to synthetic fillers. Originating from agricultural waste, plant fibers, and biomass residues, these natural materials are generally abundant in cellulose, hemicellulose, lignin, and bioactive compounds, which enhance the mechanical and thermal properties of polymer matrices while markedly increasing their biodegradability (Mohammad et al., 2024). Their hydrophilic properties facilitate water absorption, hence enhancing microbial activity and expediting decomposition. Furthermore, natural fillers frequently provide antioxidant, antibacterial, or nutrient-like properties, rendering them especially appealing for functional packaging and biomedical uses. *Sechium edule*, usually referred to as chayote, is a nutrient-dense and fiber-rich vegetable waste that has garnered interest due to its elevated cellulose and moisture-retention properties. The incorporation of *Sechium edule* into PLA matrix systems has the combined benefit of enhancing the biopolymer's strength while promoting accelerated environmental degradation, establishing it as a viable and sustainable filler for advanced biodegradable composites (García-Guzmán et al., 2022).

The primary objective of this study is to develop and characterize biodegradable composite biofilms by reinforcing polylactic acid (PLA) with *Sechium edule* peel powder, a lignocellulosic agricultural by-product, to enhance both environmental and functional performance. The importance of this work lies in addressing two pressing global challenges: the growing plastic waste crisis and the urgent need for sustainable material alternatives. By utilizing *Sechium edule* peel, underutilized natural filler, this research not only reduces the dependence on petroleum-based plastics but also valorises food industry by-products, thereby promoting circular economy practices. The necessity of such studies is underscored by the dual benefits of waste reduction and the creation of biodegradable, high-performance materials. Through comprehensive evaluation of microstructural, mechanical, moisture-interaction, and biodegradation properties, this study establishes a novel pathway for producing cost-effective, eco-friendly biofilms with strong potential for applications in green packaging, agriculture, and biomedical sectors.

## 2 Materials and biofilm fabrication

### 2.1 Sourcing and processing of *Sechium edule* for reinforcement

The peels of *Sechium edule* (commonly known as chayote), a cucurbitaceous vegetable native to Central America and widely cultivated in tropical and subtropical regions such as Mexico, Costa Rica, India, and Southeast Asia, were sourced from local agricultural markets as post-consumer vegetable waste. Taxonomically, *Sechium edule* belongs to the family Cucurbitaceae and is valued for its edible fruits, leaves, and shoots. The peels are rich in natural bioactive compounds, particularly phenolics and flavonoids, which contribute to significant antioxidant activity. These antioxidants play a critical role in neutralizing free radicals, thereby reducing oxidative stress, delaying lipid peroxidation, and enhancing the shelf life of food products when used in packaging applications. By slowing oxidative spoilage, *Sechium edule* extract-enriched biofilms may serve not only as structural materials but also as active packaging solutions. The importance of plant-derived antioxidants in the food industry is well-documented, as they offer natural alternatives to synthetic antioxidants for maintaining freshness, preventing rancidity, and extending product stability. Antioxidants sourced from plants such as phenolic acids, flavonoids, tannins, and ascorbic acid provide functional benefits in biodegradable films by imparting both protective and preservative properties, thus supporting food quality and safety (Sadighara et al., 2017).

For reinforcement use, the collected *Sechium edule* peels were carefully rinsed with distilled water to remove surface impurities, followed by oven drying at 60 °C for 24 h to eliminate residual moisture. The dried peels were mechanically ground into fine powder using a high-speed grinder and sieved to achieve uniform particle size distribution below 200 μm, ensuring consistent dispersion in the PLA matrix. The processed powder was stored in airtight containers to prevent moisture uptake prior to its incorporation. This approach not only valorises agro-waste but also aligns with circular economy principles by reducing environmental impact and improving the eco-functionality of the composite biofilms through the dual advantages of reinforcement and antioxidant-driven functional performance (Salazar-Aguilar et al., 2022).

### 2.2 PLA powder matrix

Polylactic Acid (PLA) powder was selected as the principal matrix material in this investigation due to its biodegradability, biocompatibility, and favorable thermoplastic properties. Derived from renewable resources such as corn starch and sugarcane, PLA serves as a sustainable alternative to conventional petroleum-based polymers. The PLA powder used in this study had an average particle size of less than 500 μm and was stored in airtight, moisture-free conditions to prevent premature hydrolysis, which could otherwise reduce molecular weight and compromise mechanical properties. According to the supplier datasheet, the PLA grade used exhibited a molecular weight in the range of 100,000–300,000 g/mol and a melt flow index (MFI) of 5–10 g/10 min, consistent with typical PLA grades employed for film applications. While the precise model code was not provided, these values ensured adequate flowability during solvent casting. PLA's semi-crystalline structure and relatively low glass transition temperature (Tg ≈ 55–60 °C) facilitate uniform film formation and strongly influence its mechanical and degradation behaviour. These inherent characteristics make it a suitable matrix for investigating the reinforcing effects of natural fillers such as *Sechium edule* peel powder in biofilm development (Ferrández-Montero, Lieblich, Benavente, González-Carrasco, & Ferrari, 2020; Moldovan et al., 2024).

### 2.3 Biofilm fabrication methodology

**Fig. 1:**
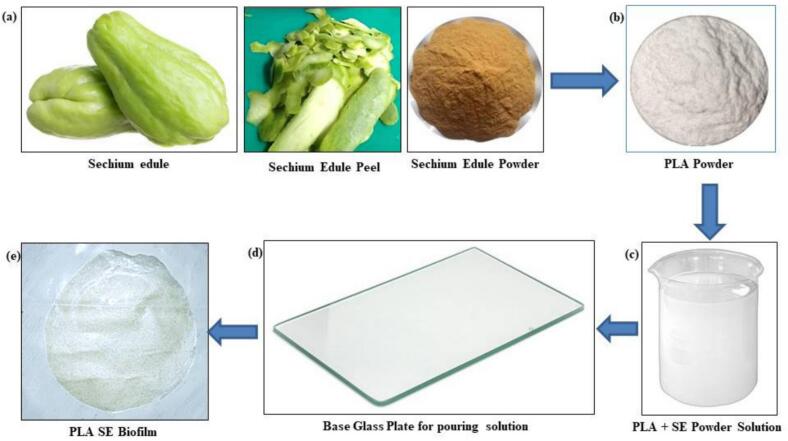
Fabrication of PLA SE Biofilm

PLA-based biofilms were fabricated using a solvent casting method, a widely recognized approach for producing homogeneous films with controlled thickness and filler distribution. PLA powder was first dissolved in chloroform at a concentration of approximately 10 % *w*/*v* to obtain a viscous and uniform solution. Processed *Sechium edule* peel powder (dried, finely ground, and sieved) was then incorporated at weight percentages of 5 %, 10 %, 15 %, and 20 % relative to the PLA matrix(Li et al., 2025; Zhang, Xu, & Bai, 2025). The PLA–SE mixtures were stirred continuously and subjected to ultrasonic treatment at 40 kHz frequency and 150 W power for 15 min to improve filler dispersion and minimize particle agglomeration. The resulting suspensions were cast onto leveled glass plates and left to dry at room temperature under a fume hood, allowing slow solvent evaporation to minimize defects such as bubbles, voids, and internal stresses—common issues in pristine PLA films. Once dried, the films were carefully peeled from the plates and subjected to secondary drying in a vacuum oven at 45 °C for 24–48 h to ensure complete removal of residual solvent. The obtained biofilms were uniform, flexible, and suitable for subsequent mechanical, thermal, morphological, and biodegradation characterizations (Balasubramanian, Aubin-Tam, & Meyer, 2019; Fazeli-Nasab et al., 2022). Fig. 1 schematically illustrates the stepwise fabrication process, including material preparation, mixing, casting, and drying of the PLA–SE composite films.

### 2.4 Composite formulation strategy and reinforcement loading details

**Fig. 2:**
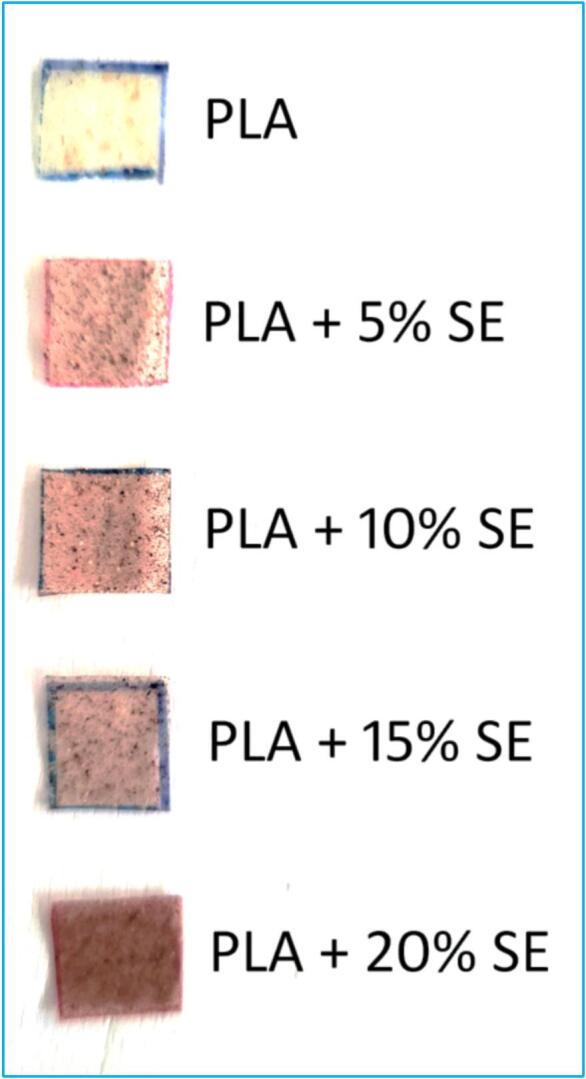
Fabricated Biofilm

PLA-based composite biofilms were developed to investigate the effect of *Sechium edule* peel powder reinforcement on their physicochemical and biodegradation properties (Kennedy, P., and Jeen, 2025; Nagendran, Kennedy, Sekar, & Pandian, 2024). PLA powder served as the matrix, while *Sechium edule* peel powder, prepared through cleaning, drying, and fine grinding, acted as the natural filler. The filler was incorporated at weight loadings of 0 % (control), 5 %, 10 %, 15 %, and 20 %, enabling systematic evaluation of its influence on film performance. The specified filler amounts were dispersed into the PLA solution under continuous stirring, with sonication applied where necessary to improve homogeneity and minimize agglomeration (Wandee et al., 2025; Zhu et al., 2024). This method ensured uniform film thickness and consistent property distribution. The dispersion quality was later confirmed through morphological and spectroscopic analyses, validating the reliability of the fabrication process (Maharaj, Vasanthanathan, Robert, Amudhan, & Nagendran, 2025). Fig. 2 illustrates the fabricated biofilms with increasing filler content (2 %, 4 %, 6 %, 8 %, and 10 %), highlighting changes in transparency, uniformity, and colour variation due to the presence of *Sechium edule* reinforcement.

## 3 Experimental methodologies

### 3.1 Microstructural and morphological characterization

#### 3.1.1 Field emission scanning electron microscopy (FESEM)

Field Emission Scanning Electron Microscopy (FESEM) was utilized to examine the surface appearance and microstructural characteristics of both clean PLA and *Sechium edule*-reinforced PLA biofilms. Before imaging, the biofilm samples were sectioned into small pieces and affixed to aluminum stubs with conductive carbon tape. To improve electrical conductivity and avert charging during imaging, the samples were coated with a thin coating of gold (about 5–10 nm in thickness) by a vacuum sputter coater. The FESEM investigation was conducted utilizing a high-resolution field emission microscope with an accelerating voltage between 5 and 10 kV. Micrographs were obtained at different magnifications to assess surface homogeneity, the dispersion of *Sechium edule* particles, and the occurrence of microvoids, fractures, or agglomerates. The morphological distinctions between plain PLA and the composites, especially regarding the distribution and interface of the natural filler within the polymer matrix, were meticulously recorded to evaluate the structural integrity and filler-matrix interaction in the produced biofilms (Guzmán-Soto et al., 2021).

#### 3.1.2 Atomic force microscopy (AFM)

Atomic Force Microscopy (AFM) was employed to examine the surface topography and nanoscale roughness of both pristine PLA and *Sechium edule*-reinforced PLA biofilms. Small film samples (about 1 cm^2^) were adhered to magnetic sample discs and analyzed using a tapping mode AFM configuration to prevent damage to the delicate polymer surfaces. Measurements were conducted under ambient conditions utilizing a silicon cantilever with a resonance frequency of approximately 300 kHz and a tip radius of less than 10 nm. Surface scans were conducted over regions of 5 μm × 5 μm and 10 μm × 10 μm to assess morphological characteristics including surface roughness (Ra), particle distribution, and textural alterations induced by the filler. The AFM program assessed roughness characteristics, indicating that the incorporation of *Sechium edule* elevated surface roughness, with Ra values increasing from around 38.6 nm for pristine PLA to 92.4 nm for 20 % filler loading. The nanoscale features offered insights into the homogeneity of filler dispersion and facilitated further examination of moisture interaction and degrading behaviour of the biofilms (Kennedy, Raghav, Britto, & Amudhan, 2025).

### 3.2 Chemical interaction and bonding

#### 3.2.1 Fourier transform infrared spectroscopy (FTIR)

Fourier Transform Infrared Spectroscopy (FTIR) was utilized to examine the chemical structure of both virgin and *Sechium edule* peel powder-reinforced PLA biofilms. The observations were performed with an FTIR spectrometer featuring an Attenuated Total Reflectance (ATR) accessory, facilitating direct surface investigation of solid films without additional sample preparation. Spectra were acquired within the range of 4000 to 500 cm^−1^ at a resolution of 4 cm^−1^, averaging 32 scans per sample to enhance signal clarity. Biofilm samples incorporating 0 %, 5 %, 10 %, 15 %, and 20 % *Sechium edule* powder were examined to identify the functional groups and evaluate the chemical interactions within the composite matrix. All films were evaluated under uniform settings to provide uniformity and comparability among the different formulations (Crisp et al., 2023).

#### 3.2.2 UV–Visible spectroscopy for optical properties

UV–Visible spectroscopy was utilized to assess the optical characteristics of PLA biofilms, encompassing both pristine and *Sechium edule* peel powder-reinforced formulations. The analysis utilized a UV–Visible spectrophotometer within the wavelength range of 200 to 800 nm. Rectangular film samples were meticulously cut to a consistent size and positioned directly in the trajectory of the laser beam without additional treatment. Measurements were conducted in transmittance mode, utilizing air as the reference standard. All biofilm samples (0 %, 5 %, 10 %, 15 %, and 20 % SE content) were scanned using identical parameters to provide uniform comparison. The acquired data were utilized to evaluate the impact of natural filler incorporation on the light transmission and transparency of PLA sheets (Alidokht et al., 2024).

### 3.3 Mechanical and functional properties

#### 3.3.1 Tensile testing

Tensile testing was performed to assess the mechanical strength and flexibility of both pristine and *Sechium edule* peel powder-reinforced PLA biofilms. The evaluations were conducted utilizing a universal testing machine (UTM) in compliance with ASTM D882, which delineates the standard methodology for assessing thin plastic films. Rectangular specimens were meticulously excised from the cast biofilms, measuring 100 mm in length and 10 mm in width. Before testing, all samples were acclimatized at room temperature (23 ± 2 °C) and 50 ± 5 % relative humidity for 48 h. A uniform crosshead velocity of 5 mm/min was sustained throughout the experiment, with the gauge length established at 50 mm. A minimum of five specimens from each formulation (0 %, 5 %, 10 %, 15 %, and 20 % *Sechium edule* loading) were evaluated to guarantee statistical reliability. The stress-strain data were collected to ascertain characteristics including tensile strength, elongation at break, and Young's modulus (Kennedy, V., and A., 2024; Kennedy, Jeen Robert, Prince, & R., Hikku, G. S., & Kaliraj, M., 2024).

#### 3.3.2 Water absorption and swelling behaviour

The water absorption and swelling behaviour of PLA biofilms, both unmodified and supplemented with *Sechium edule* peel powder, were assessed to evaluate their hydrophilicity and moisture interaction properties. The examinations were performed in accordance with ASTM D570 norms. Biofilm samples were sectioned into homogeneous rectangles, and their initial dry weight (W₀) was documented following desiccation in a vacuum oven at 50 °C for 24 h. The samples were thereafter submerged in distilled water at ambient temperature (25 ± 2 °C) for a predetermined duration (generally 24 h). Following immersion, surplus surface water was delicately removed with blotting paper, and the ultimate swelled weight (Wₜ) was recorded. The percentage of water absorption and swelling was determined by the weight differential. All measurements were conducted in triplicate for each biofilm composition to guarantee reproducibility and precision (Waqas, Farhan, Haider, Qumar, & Raza, 2023).

#### 3.3.3 Biodegradability assessment under controlled conditions

The biodegradability of PLA biofilms, both unaltered and fortified with *Sechium edule* peel powder, was assessed under regulated composting settings to replicate natural degradation environments. The assessment adhered to the ISO 14855 standard procedure, which quantifies aerobic biodegradability by measuring the carbon dioxide (CO₂) produced. Rectangular film samples (about 2 cm × 2 cm) were entombed in a compost medium consisting of soil, cow dung, and organic waste in a weight ratio of 2:1:1, sustained at a constant temperature of 58 ± 2 °C and 50–60 % relative humidity. The samples were systematically extracted at predetermined intervals, gently cleaned to eliminate adherent soil, dried in a vacuum oven at 50 °C, and weighed to assess the weight loss. Each test was conducted in triplicate to guarantee precision and reproducibility (Delacuvellerie et al., 2021).

## 4 Results and discussion

### 4.1 Microstructural Insights from FESEM and AFM

#### 4.1.1 Reinforcement dispersion and interfacial adhesion

**Fig. 3:**
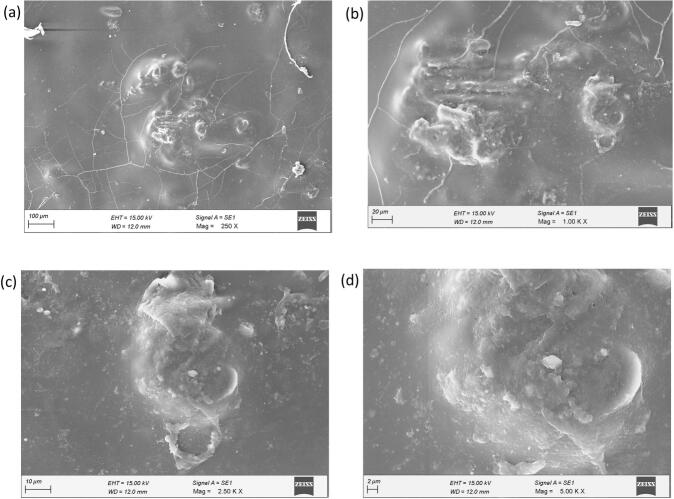
FESEM Analysis of PLA Biofilm.

The FESEM investigation of the PLA biofilm offers essential insights into its inherent morphology, emphasizing significant structural flaws that affect its mechanical and barrier properties. At low magnification (Fig. 3.a), the film displays a continuous but significantly fractured surface, characterized by uneven rough patches interspersed throughout. The extensive fissures indicate internal tensions, perhaps caused by rapid solvent evaporation during film casting, signifying the film's inherent brittleness. At intermediate magnification (Fig. 3.b), the rough features become more prominent, with cracks emanating from or encircling these areas, affirming that these irregular regions act as stress concentration locations. Upon meticulous examination at elevated magnification (Fig. 3.c), the demarcation between the coarse patches and the smoother PLA matrix is stark, with the rough regions exhibiting granular textures, indicative of localized phase separation or polymer aggregation. At maximum magnification (Fig. 3.d), the irregular surfaces exhibit micro-voids and a pitted appearance, substantiating the existence of microstructural flaws that could undermine barrier and mechanical integrity. The FESEM results indicate that the plain PLA biofilm has a heterogeneous structure characterized by brittle fracture zones, surface roughness, and porosity. The defects restrict the material's use in load-bearing or barrier-critical applications, underscoring the necessity for process optimization techniques such as controlled solvent evaporation, thermal annealing, or plasticizer addition to improve its structural uniformity and performance.

**Fig. 4:**
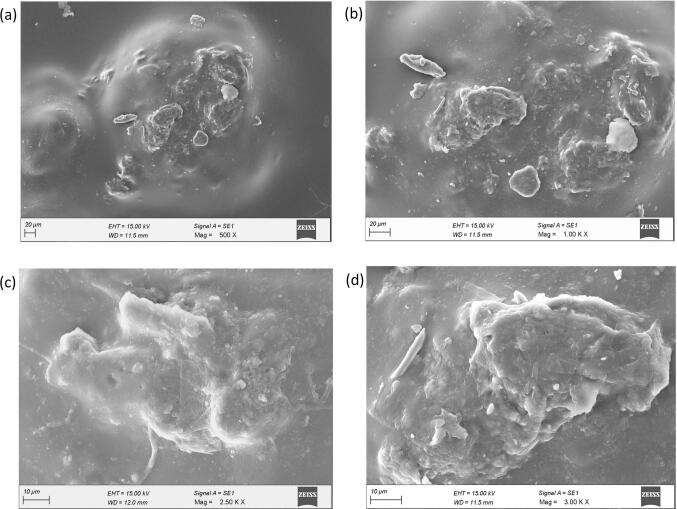
FESEM Analysis of PLA Biofilm with 6 % SE

The FESEM investigation demonstrates significant morphological integrity in PLA biofilms reinforced by 6 % *Sechium edule* particles. Fig. 4(a) at low magnification (500×), the reinforced biofilm displays a smoother and more cohesive surface with negligible breaking, in sharp contrast to the unmodified PLA, which reveals extensive fracture networks. The scattered and embedded *Sechium edule* particles are distinctly observable, signifying effective incorporation into the PLA matrix. Fig. 4(b) with intermediate magnification (1.00KX) demonstrates that these particles retain their uneven, natural form despite being well enclosed by the PLA. Significantly, even in areas with bigger agglomerates, there is no indication of crack starts or stress localization, underscoring enhanced interfacial adhesion. Fig. 4(c) with enhanced magnification (2.50KX) reveals a close adhesion between the PLA and the coarse, fibrous texture of a *Sechium edule* aggregate, with no discernible voids or delamination, thereby substantiating the concept of robust matrix-filler compatibility. Fig. 4(d) at maximum magnification (3.00KX), the junction between the filler and matrix is continuous and devoid of defects, hence affirming the composite's structural integrity. The matrix tightly aligns with the intricate structure of the filler, guaranteeing homogeneous stress distribution. The morphological enhancements—characterized by less cracking, improved dispersion, and higher interface quality—clearly suggest enhanced mechanical and barrier capabilities of the reinforced biofilm relative to unmodified PLA. The findings endorse the viability of *Sechium edule*-reinforced PLA as a structurally dependable and functionally superior biodegradable material.

#### 4.1.2 Surface roughness and particle integration

**Fig. 5:**
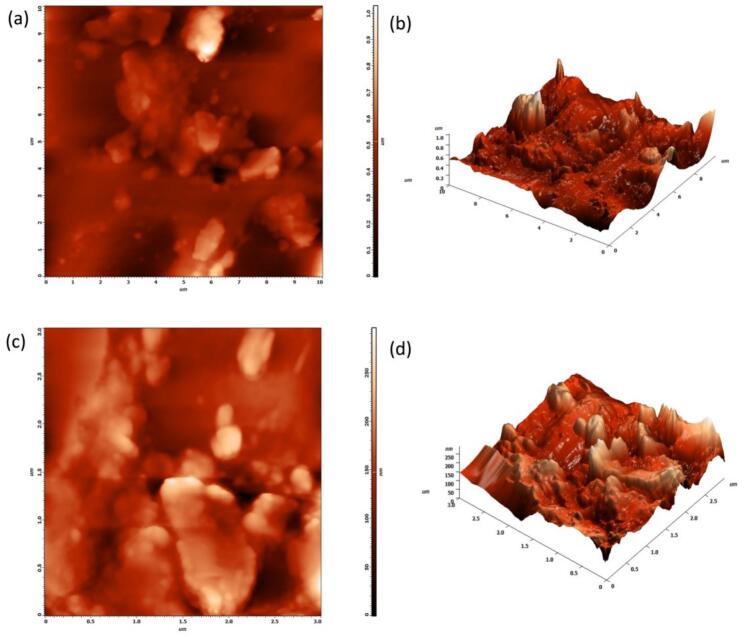
AFM Analysis (a & b) PLA Biofilm (c & d) PLA Biofilm reinforced with 6 % SE.

The atomic force microscopy (AFM) research offers comprehensive insights into the nanoscale surface morphology of the plain PLA biofilm, emphasizing significant topographical abnormalities and heterogeneity. Fig. 5 (a), the two-dimensional height map of a 10 μm × 10 μm scan area, illustrates a markedly rough and irregular surface, featuring numerous brilliant peaks and dark valleys that correspond to protrusions and depressions reaching around 800 nm in height. The unevenly formed surface aggregates indicate phase separation or unequal polymer precipitation following solvent evaporation. Fig. 5(b), the three-dimensional topographic representation of the same region, substantiates this interpretation by visually illustrating a rocky, undulating surface with significant peak-to-valley discrepancies, closely approximating mountainous terrain. This pronounced nanoscale roughness is unusual for uniformly cast polymer films and corresponds with the discontinuities and surface fractures evident in the FESEM pictures. This surface heterogeneity is expected to adversely impact the film's optical clarity, mechanical properties, and moisture barrier performance. The identified faults suggest inadequate film formation, potentially resulting from fast or unregulated solvent evaporation, insufficient polymer chain organization, or surface precipitation of oligomers or contaminants. The AFM results indicate that the plain PLA biofilm exhibits a significantly defective and morphologically uneven surface, underscoring the necessity for enhanced processing conditions to produce smoother and more structurally coherent films.

The AFM evaluation of the *Sechium edule*-reinforced PLA biofilm demonstrates significantly improved nanoscale surface morphology relative to the unmodified PLA film, indicating enhanced uniformity and less roughness. Fig. 5(c) presents a 2D height map of a 3 μm × 3 μm scan area, illustrating discrete embedded particles recognized as *Sechium edule* reinforcement, uniformly dispersed inside the PLA matrix. These particles display irregular but regulated protrusions, whilst the surrounding polymer matrix seems more uniform and homogenous compared to the rough, aggregated surface of standard PLA. The highest surface height fluctuation is restricted to approximately 280 nm, considerably less than the approximately 800 nm observed in the unreinforced film. The diminished roughness signifies a better regulated film production process, wherein the reinforcement not only integrates well but also mitigates the random polymer aggregation that resulted to surface imperfections in unmodified PLA. Fig. 5(d), the three-dimensional topographic representation, corroborates these findings by depicting elevated structures associated with the reinforcement, arising from a predominantly flat substrate. The vertical height range is limited to around 280 nm, confirming that topographical differences result from functional reinforcement rather than uncontrolled matrix roughness. The enhancements in surface regularity augment the film's potential efficacy by diminishing light scattering, facilitating uniform surface interactions, and reducing stress concentrations. The AFM investigation confirms that *Sechium edule* reinforcement markedly improves the surface quality and structural integrity of PLA biofilms, rendering them more appropriate for applications requiring enhanced mechanical, optical, and interfacial features.

### 4.2 Molecular interaction and functional group shift (FTIR & UV–Vis)

#### 4.2.1 Confirmation of bonding and structural shifts

**Fig. 6:**
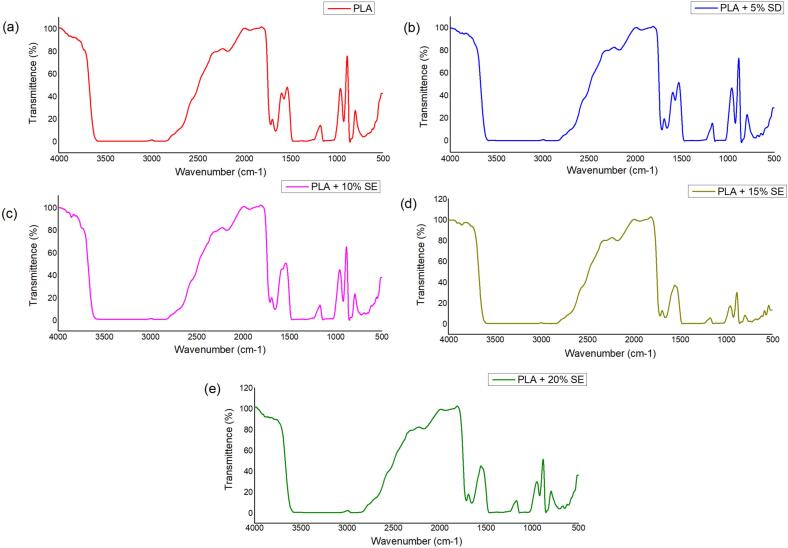
FTIR Analysis

The FTIR spectra of PLA and PLA/SE composites (Fig. 6) are presented in transmittance mode to illustrate the functional group interactions and filler incorporation within the biofilm matrix. The neat PLA spectrum (Fig. 6a) displayed characteristic absorption bands near 1750 cm^−1^ (C=O stretching of ester carbonyl), 2990–2940 cm^−1^ (C—H asymmetric and symmetric stretching), and 1180–1080 cm^−1^ (C–O–C stretching of ester linkages), confirming the identity of unmodified PLA. With the incorporation of 5 % filler (Fig. 6b), a broad band appeared in the region of ∼3300 cm^−1^, associated with O—H stretching vibrations, indicating the presence of hydroxyl-rich lignocellulosic constituents. This band became progressively more intense with increasing SE content from 10 % to 20 % (Figs. 6c–e), reflecting the higher concentration of hydrophilic functional groups contributed by the natural filler. Additional bands in the regions 1600–1500 cm^−1^ and 1240–1030 cm^−1^ became more distinct with increasing SE concentration, which are attributable to cellulose and lignin vibrations. Importantly, the major PLA peaks remained at their original positions, suggesting that the interaction between PLA and SE is primarily physical rather than chemical. The observed growth in the O—H stretching region is consistent with the enhanced hydrophilicity, water absorption, and biodegradability of the composites, while the reinforcement of lignocellulosic bands confirms the gradual incorporation and uniform dispersion of SE powder within the PLA matrix.

#### 4.2.2 Impacts on photodegradability and optical transparency

**Fig. 7:**
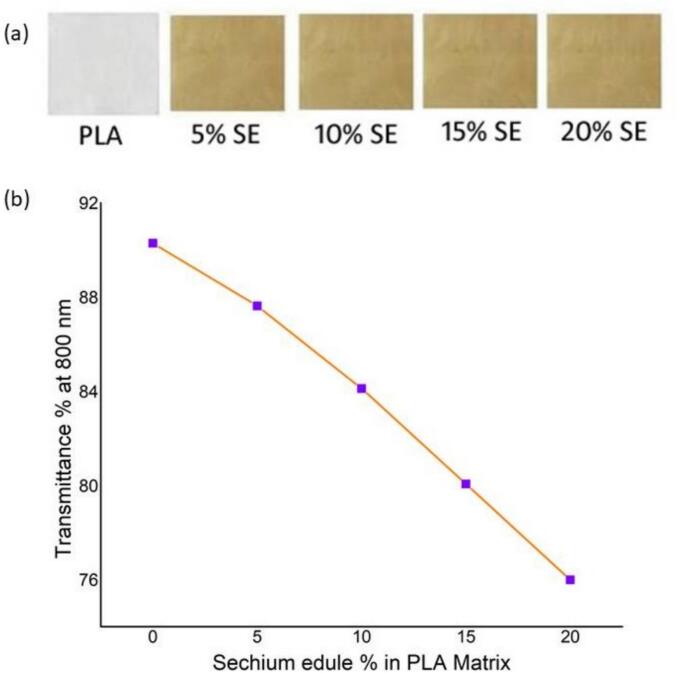
**(a)** UV Photographs of PLA/SE Biofilms (b) UV Visible testing results.

Fig. 7(a) illustrates a macroscopic visual comparison between the simple PLA biofilm and PLA biofilms augmented by escalating concentrations of *Sechium edule* (5 %, 10 %, 15 %, and 20 %). The unadorned PLA sheet exhibits significant transparency and whiteness, signifying its excellent intrinsic optical clarity. The *Sechium edule*-reinforced films exhibit a gradual colour transition from pale yellow to richer ochre tones, which become more pronounced with higher filler concentrations. This hue is ascribed to the inherent pigments found in *Sechium edule*, functioning as intrinsic colorants. A concomitant reduction in transparency is evident, indicating heightened light scattering attributable to the particle characteristics of the reinforcement. The qualitative alterations are essential for comprehending the trade-offs associated with the incorporation of natural fillers into biopolymer matrices and offer visual evidence of the integration of plant-derived elements inside the PLA film.

Fig. 7(b) presents a quantitative assessment of light transmittance at 800 nm across the identical series of biofilms. The transmittance values exhibit a consistent decrease from approximately 90 % for plain PLA to around 76 % for PLA containing 20 % *Sechium edule*. This nearly linear reduction aligns closely with eye observations and is mostly attributable to enhanced light scattering at the interfaces between PLA and *Sechium edule* particles, which possess varying refractive indices. Furthermore, negligible absorption by natural pigments may also influence the reported trend. These findings indicate that whereas *Sechium edule* improves structural characteristics (as evidenced by FESEM and AFM studies), it diminishes optical clarity. This knowledge is vital for applications such as food packaging or biomedical films, where transparency is essential, requiring a compromise between mechanical enhancement and visual performance.

### 4.3 Mechanical enhancement by *Sechium edule*

**Fig. 8:**
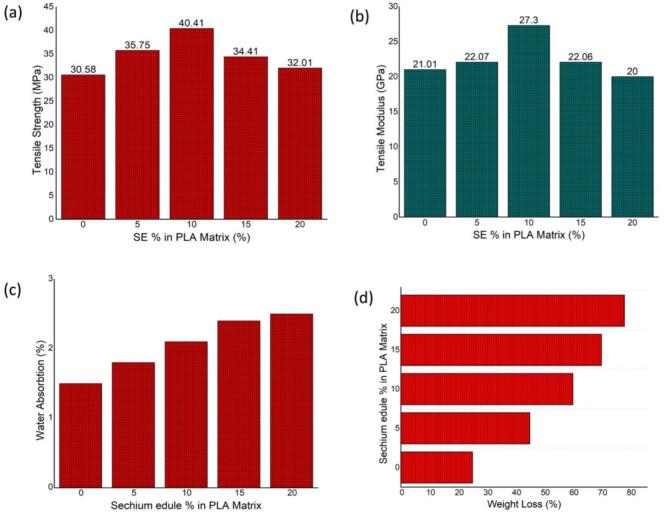
(a,b) Tensile test results (c) Water absorption test results (d) Biodegradation test results

Fig. 8(a) depicts the fluctuation in tensile strength of PLA biofilms augmented with escalating concentrations of *Sechium edule* (SE) filler, from 0 % to 20 %. The tensile strength of pure PLA is recorded as 30.58 MPa. The inclusion of SE filler results in a significant enhancement, with strength rising to 35.75 MPa at 5 % SE and reaching a maximum of 40.41 MPa at 10 % SE. This improvement is due to the reinforcing capacity of SE particles, which, being inherently hard and cellulose, efficiently distribute the tensile load when adequately scattered in the PLA matrix. FESEM study (Fig. 4) corroborates this by demonstrating robust interfacial adhesion and no micro-cracking, in contrast to the plain PLA (Fig. 3). At 15 % and 20 % SE, the tensile strength decreases to 34.41 MPa and 32.01 MPa, respectively, as a result of particle agglomeration, inadequate matrix wetting, and interrupted polymer continuity, which facilitate stress concentration and impede stress transfer.

Fig. 8(b) similarly illustrates the difference in tensile modulus, indicative of stiffness, across the identical SE concentrations. The modulus rises from 21.01 MPa (0 % SE) to 22.07 MPa (5 % SE), with a peak of 27.3 MPa at 10 % SE, underscoring the effective limitation of polymer chain mobility resulting from filler incorporation. Exceeding 10 %, the modulus decreases to 22.06 MPa (15 % SE) and further diminishes to 20 MPa (20 % SE), suggesting that at elevated filler concentrations, agglomeration and inadequate filler-matrix interactions undermine the stiffening advantages of SE. Significantly, at 20 % SE, the modulus decreases below that of pristine PLA, indicating filler saturation and diminished material integrity.

The mechanical findings correlate well with morphological observations from FESEM and AFM. PLA films demonstrate considerable surface imperfections and elevated nanoscale roughness (Fig. 3, Fig. 5), which correspond to their diminished tensile characteristics. Conversely, SE-reinforced films, particularly at a 10 % loading, exhibit enhanced structural integrity, homogeneous dispersion, and a smoother morphology (Fig. 4, Fig. 6), resulting in higher mechanical performance. The findings indicate that a 10 % SE loading is best for enhancing tensile strength and stiffness while maintaining film integrity. This work illustrates the potential of SE as natural filler to enhance PLA biofilms and indicates that surface modification or dispersion strategies may further augment performance at elevated filler concentrations.

### 4.4 Hydrophilicity and water uptake behaviour

#### 4.4.1 Water absorption vs reinforcement content

The water absorption behaviour of PLA biofilms reinforced with different concentrations of *Sechium edule* (SE) is illustrated in Fig. 8(c). The findings indicate a distinct, concentration-dependent escalation in water absorption, commencing at roughly 1.5 % for pure PLA (0 % SE) and advancing to nearly 2.6 % with 20 % SE incorporation. This trend is mainly due to the hydrophilic characteristics of *Sechium edule*, lignocellulosic filler abundant in hydroxyl groups that may establish hydrogen bonds with water molecules. The augmented presence of SE inside the PLA matrix improves the overall hydrophilicity of the composite films, thereby facilitating water absorption. Evidence from FESEM and AFM pictures demonstrates that the filler particles contribute to surface roughness and may generate interfacial micro-voids, especially at elevated SE concentrations, which serve as additional locations for water penetration. Moreover, the aggregation of SE at elevated loadings (15–20 %) may compromise the continuity of the PLA matrix, hence promoting moisture transport through the film. This enhanced water absorption may expedite biodegradation, but it also undermines dimensional stability and mechanical integrity in humid conditions, as seen by the reduction in tensile strength exceeding 10 % SE. Therefore, meticulous tuning of SE content is crucial to reconcile reinforcing advantages with water sensitivity, particularly for uses in biodegradable packaging or regulated environmental settings.

#### 4.4.2 Structural reasons for moisture interaction

The increased moisture interaction in *Sechium edule* (SE)-reinforced PLA biofilms is structurally influenced by the hydrophilic characteristics and morphological attributes of the filler. FESEM research indicated that SE particles exhibit uneven geometries and surface roughness, resulting in micro-voids and interfacial gaps inside the PLA matrix, particularly at elevated loadings (15 % and 20 %). These structural defects function as capillary vessels, enabling water ingress. AFM measurements corroborated this, indicating a substantial increase in surface roughness (Ra) from 28.3 nm in pristine PLA to 65.7 nm at 20 % SE. The augmented roughness amplifies the surface area available to moisture, facilitating water adhesion and absorption. Furthermore, the cellulose composition of SE has a significant density of hydroxyl (-OH) groups that can establish hydrogen bonds with water molecules. Consequently, water absorption rose from 1.5 % in unmodified PLA to 2.6 % at 20 % SE, demonstrating a significant relationship between microstructural disturbances and higher moisture absorption in the composite films.

### 4.5 Biodegradability performance

#### 4.5.1 Breakdown rate and environmental behaviour

Fig. 8(d) illustrates the weight loss % of PLA biofilms as the concentrations of *Sechium edule* (SE) increase, offering a direct assessment of their biodegradability. The data indicate a significant and steady rise in weight loss, from roughly 25 % for pure PLA to 78 % for 20 % SE content, demonstrating a robust positive association between SE loading and degradation rate. This improvement in degradability is due to various synergistic causes. The hydrophilic characteristics of *Sechium edule*, abundant in hydroxyl groups, enhance water absorption, hence inducing swelling and enabling microbial and enzymatic penetration into the PLA matrix. Moreover, as a natural and biodegradable substance, SE not only decomposes rapidly but also generates internal gaps and microchannels during its degradation, so expediting the disintegration of the adjacent polymer. The organic composition may function as a nutrition source, promoting microbial colonization and enzymatic activity on the film surface. Microstructural analyses using FESEM and AFM indicate that the roughness and morphology resulting from SE inclusion enhance the surface area available to degrading agents. These findings underscore the potential of SE as functional filler that not only enhances but also markedly improves the environmental performance of PLA, providing a customizable method for designing biodegradable materials appropriate for short-life-cycle applications and environmentally responsible disposal.

#### 4.5.2 Correlation between microstructure and degradability

The microstructural features identified via FESEM and AFM investigations demonstrate a distinct relationship with the improved degradability of *Sechium edule* (SE)-reinforced PLA biofilms. As the SE concentration escalated from 0 % to 20 %, the micrographs demonstrated a shift from a comparatively smooth and dense PLA matrix to an increasingly coarse surface featuring embedded, irregular SE particles. At elevated filler loadings (15 % and 20 %), discernible interfacial gaps and porous areas surrounding SE agglomerates became evident, possibly facilitating enhanced water infiltration and microbiological ingress. This morphological evolution corresponds with the biodegradability findings, indicating that weight loss escalated from 25 % in pure PLA to 78 % with 20 % SE concentration. The AFM pictures indicated an increase in surface roughness (Ra), rising from 28.3 nm for pure PLA to 65.7 nm at 20 % SE, suggesting a more conducive topography for microbial colonization and enzymatic degradation. The compromised matrix integrity and augmented surface area linked to elevated SE content directly promoted hydrolytic and microbial degradation, thereby establishing a robust structure–property–function relationship between filler-induced microstructural alterations and the observed increase in degradation rate.

## 5 Sustainable implications and potential applications

### 5.1 Green packaging, agricultural films, or biomedical uses

The engineered PLA biofilms augmented with *Sechium edule* peel powder demonstrate considerable potential for use in sustainable packaging, agricultural films, and specific biomedical fields. The improved biodegradability (about 78 % weight loss at 20 % SE concentration) and the natural origin of the filler correspond with contemporary global aspirations for environmentally sustainable alternatives to petroleum-derived plastics. The intermediate mechanical integrity achieved at optimal filler loading (5–10 %) and the enhanced moisture interaction from hydrophilic functional groups render the films appropriate for short-lifecycle food packaging when moisture permeability is acceptable or advantageous. In agriculture, these films can function as biodegradable mulching sheets or seed covers, hence minimizing the necessity for collection and disposal after usage. The lack of hazardous residues and the biocompatibility of both PLA and *Sechium edule* render the composite a viable candidate for low-risk biomedical applications, including temporary wound dressings or disposable surgical drapes. These applications highlight the composite's sustainable lifecycle and its conformity with circular economy principles.

### 5.2 Life-cycle analysis perspectives

From a life-cycle analysis (LCA) perspective, incorporating *Sechium edule* peel powder into PLA biofilms strengthens sustainability by repurposing agricultural waste and reducing reliance on virgin polymers. As a biodegradable and renewable resource, *Sechium edule* lowers the environmental burden associated with raw material extraction and end-of-life disposal. Compared with petroleum-based plastics, the composite films exhibit reduced carbon emissions across their life cycle, from sourcing to degradation. Their biodegradability, with up to 78 % weight loss under controlled conditions, ensures faster breakdown and minimizes landfill accumulation. Furthermore, the optimized solvent casting method can be adapted into low-energy production processes, further reducing ecological impact. By valorising agro-waste and aligning with circular economy principles, these biofilms offer an environmentally advantageous alternative for applications in packaging, agriculture, and related sectors.

### 5.3 Scalability of Sechium edule-reinforced PLA biofilms

The scalability of *Sechium edule*-reinforced PLA biofilms is encouraging owing to the accessibility of raw ingredients, ease of manufacture, and alignment with current industrial processes. *Sechium edule* (chayote) is extensively cultivated and produces significant peel waste, which can be responsibly procured in large quantities from agro-industrial sectors. The reinforcing powder can be generated using scalable methods like drying and milling, whilst the solvent casting methodology utilized in this research can be modified for roll-to-roll or extrusion film production processes for larger-scale manufacturing. The reinforcement loadings employed (5–20 wt%) are within practical ranges that do not impede processability, suggesting that minimum adjustments are required in industrial contexts. The biofilm formulation employs biodegradable, non-toxic materials, thereby minimizing regulatory limitations and expediting approval for commercial purposes, including packaging and disposable biomedical items. These characteristics collectively endorse the economic and technical feasibility of scaling this composite material for extensive sustainable implementation.

## 6 Conclusion

This study demonstrates that incorporating *Sechium edule* peel powder into PLA yields measurable improvements in key functional properties up to an optimal filler loading. Mechanically, tensile strength increased from 30.58 MPa (0 % SE) to a peak of 40.41 MPa at 10 % SE (≈32 % increase), while tensile modulus rose from 21.01 MPa to 27.30 MPa at the same loading (≈30 % increase). Beyond 10 % SE the mechanical benefits declined (e.g., tensile strength 34.41 MPa at 15 % and 32.01 MPa at 20 %), consistent with agglomeration and matrix disruption observed in microscopy. Hydrophilicity and biodegradability were similarly affected by filler content: water absorption increased from ∼1.5 % (plain PLA) to ∼2.6 % at 20 % SE (≈73 % increase), and weight-loss under controlled composting rose from ∼25 % to ∼78 % (≈3.1×). FTIR confirmed progressive incorporation of hydroxyl-rich lignocellulosic components, and FESEM/AFM showed improved film continuity and filler–matrix contact at optimal loadings—together these data explain the trade-off between enhanced biodegradability and altered moisture/optical behaviour.

While the results are encouraging, conclusions are stated with appropriate restraint: improvements are formulation- and process-dependent and decline at high filler loadings due to dispersion limits. Key limitations include the laboratory-scale solvent-casting method, the short-term controlled biodegradation test (not full environmental variability), and the need for rheological/compatibilizer optimization for melt-processing. Future work should focus on surface treatment or compatibilizer to reduce agglomeration at higher loadings, pilot-scale extrusion/roll-to-roll trials and rheological tuning to assess industrial processability, extended real-environment biodegradation and life-cycle analysis to validate environmental benefits, and application-specific testing (barrier properties, cytocompatibility for biomedical uses). These steps will clarify the practical potential of *Sechium edule*-reinforced PLA for sustainable packaging, agricultural films, and selected biomedical applications without overstating readiness for immediate large-scale deployment.

## CRediT authorship contribution statement

**Kaliraj Medadurai:** Writing – review & editing, Methodology, Investigation, Data curation, Conceptualization. **Senthil Maharaj Kennedy:** Writing – original draft, Software, Methodology, Investigation, Conceptualization. **Jegan Balasubramani:** Visualization, Validation, Supervision, Investigation. **Selvi Shanmugavelayutham:** Methodology, Investigation, Formal analysis, Data curation.

## Declaration of competing interest

The authors declare that they have no known competing financial interests or personal relationships that could have appeared to influence the work reported in this paper.

## Data Availability

No data was used for the research described in the article.

## Appendix A Supplementary data

Supplementary material 1 Image 1

Supplementary material 2 Image 2

Supplementary material 3 Image 3

Supplementary material 4 Image 4

Supplementary material 5 Image 5

Supplementary material 6 Image 6
