# Supplementary figures and images for: Microstructural and functional analysis of PLA-based biofilm reinforced with Sechium edule
Source: Food Chem X. 2025 Oct 3;31:103130. doi: 10.1016/j.fochx.2025.103130 (PMC12549793; doi:10.1016/j.fochx.2025.103130)

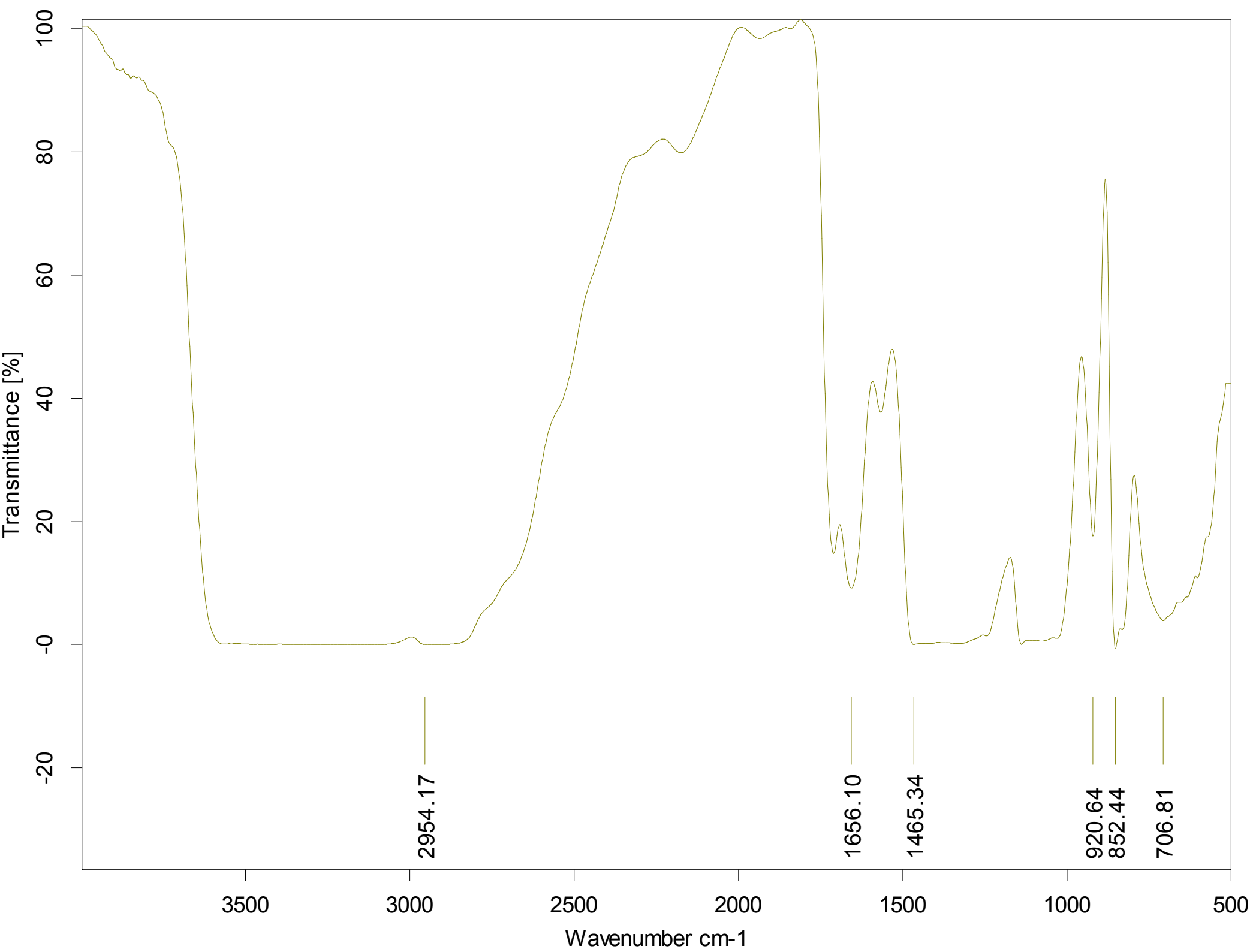

Supplement: Supplementary file 1 — Supplementary material 1 [file mmc1.pdf]

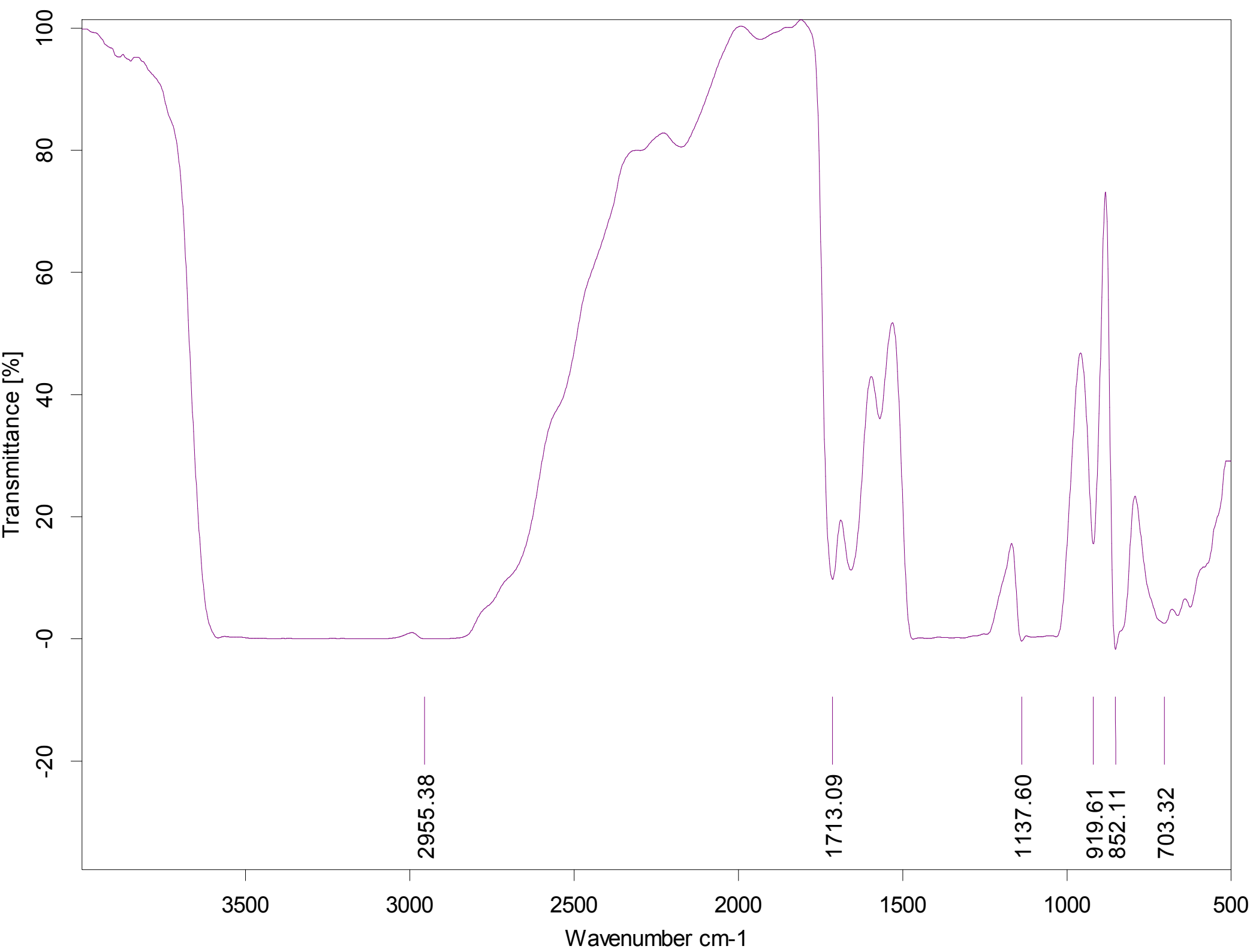

Supplement: Supplementary file 2 — Supplementary material 2 [file mmc2.pdf]

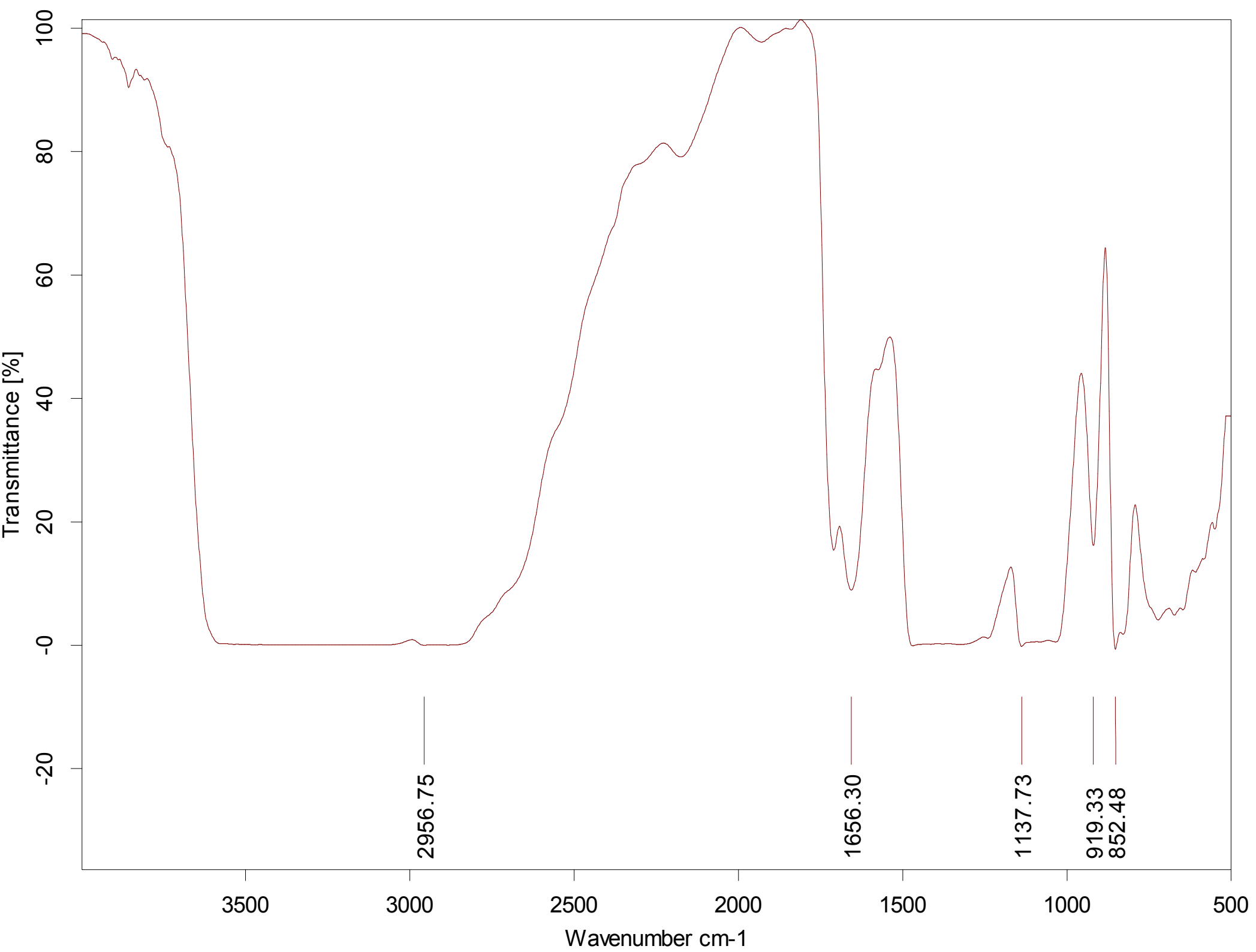

Supplement: Supplementary file 3 — Supplementary material 3 [file mmc3.pdf]

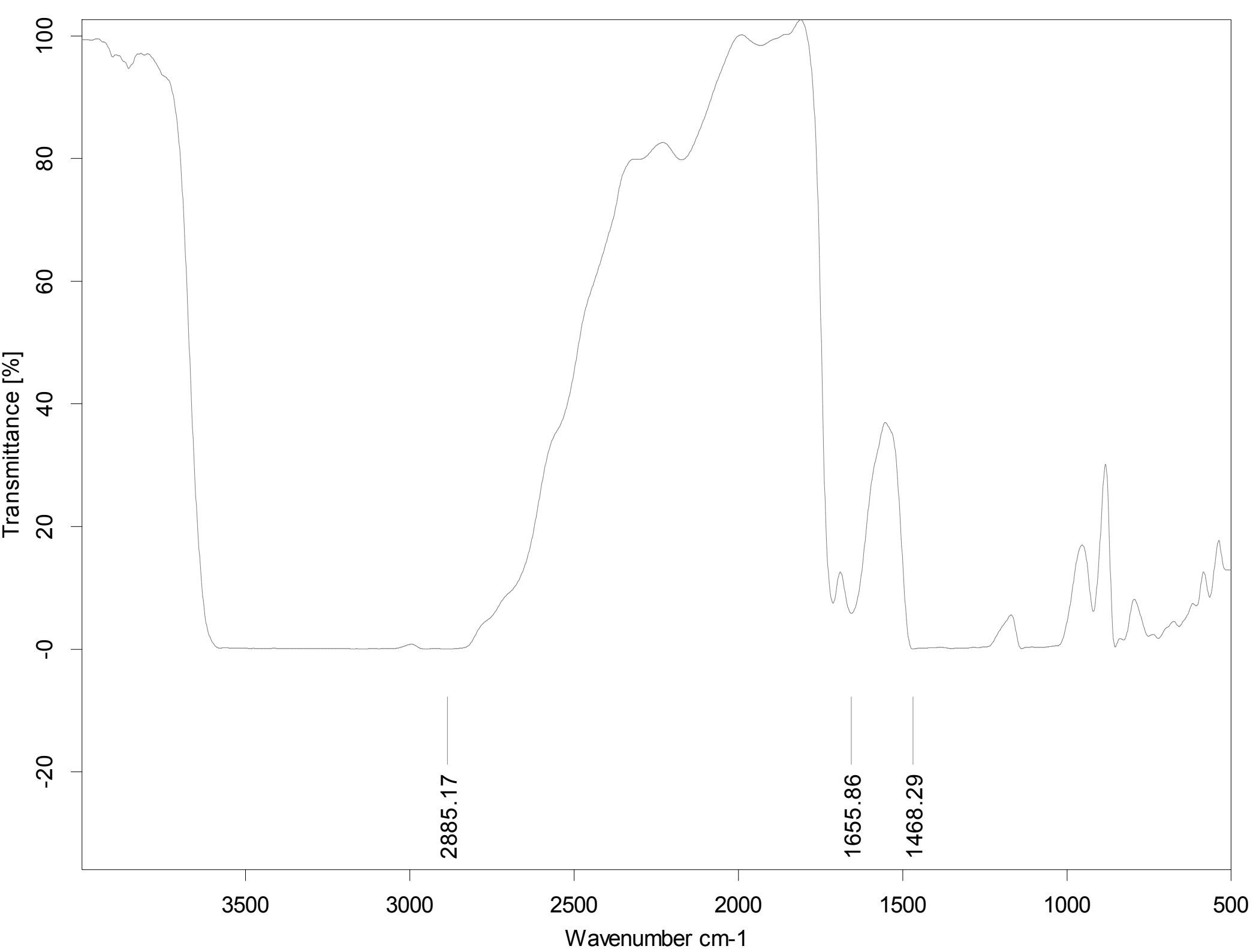

Supplement: Supplementary file 4 — Supplementary material 4 [file mmc4.pdf]

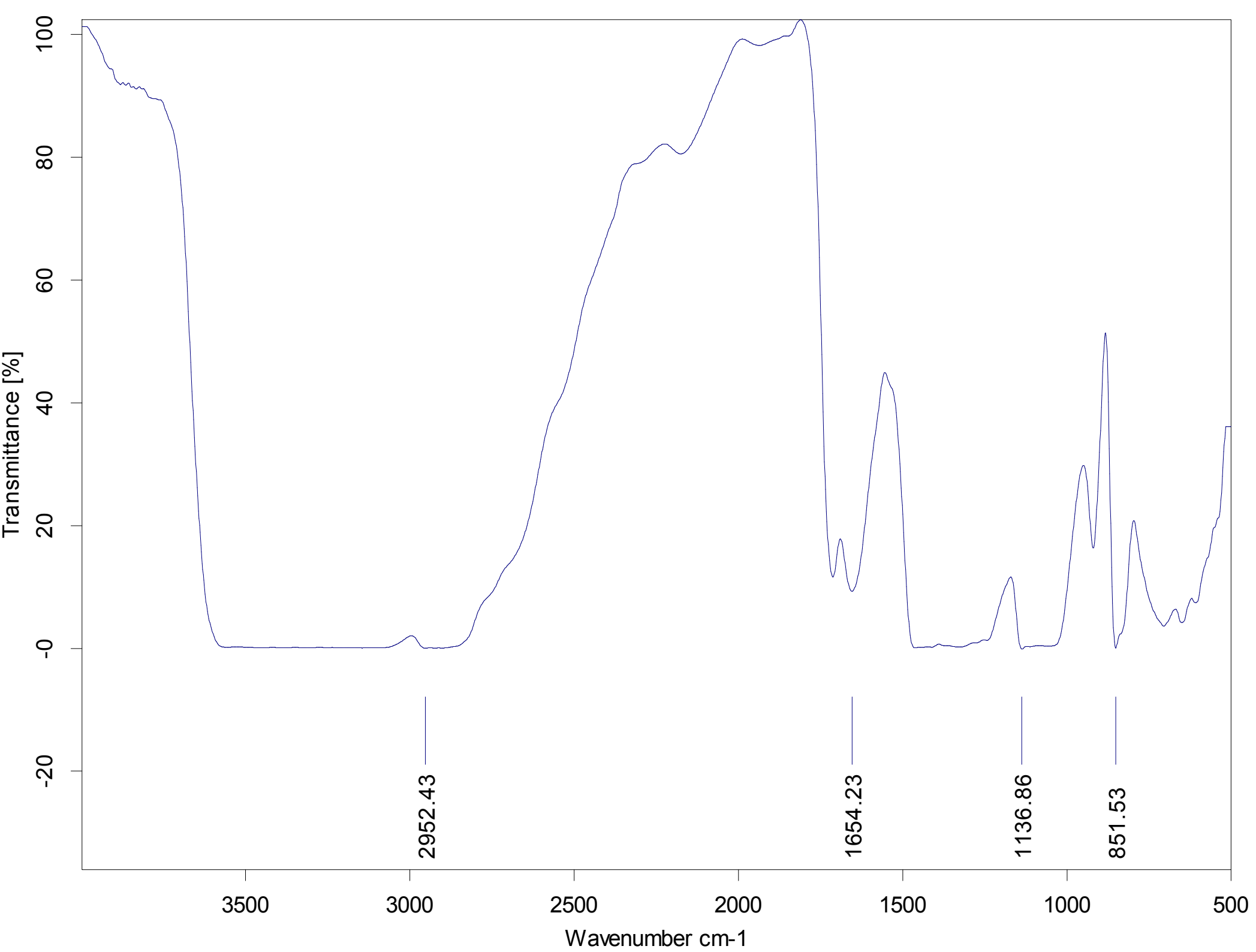

Supplement: Supplementary file 5 — Supplementary material 5 [file mmc5.pdf]
